# Exploring tumorigenesis as a process of phenotypic state-space exploration and selective funneling

**DOI:** 10.1093/emph/eoag022

**Published:** 2026-09-22

**Authors:** Frédéric Thomas, Michael L Wong, Robert M Hazen, Antoine M Dujon, Klara Asselin, Jordan Meliani, Beata Ujvari, Mario Campone, Pascal Pujol, Catherine Alix-Panabières, Jean-Pascal Capp, Aurora M Nedelcu

**Affiliations:** CREEC/CANECEV, MIVEGEC (CREES) Department, University of Montpellier, CNRS, IRD, Montpellier, France; Earth and Planets Laboratory, Carnegie Institution for Science, Washington, DC, USA; Earth and Planets Laboratory, Carnegie Institution for Science, Washington, DC, USA; CREEC/CANECEV, MIVEGEC (CREES) Department, University of Montpellier, CNRS, IRD, Montpellier, France; School of Life and Environmental Sciences, Deakin University, Waurn Ponds, VIC, Australia; CREEC/CANECEV, MIVEGEC (CREES) Department, University of Montpellier, CNRS, IRD, Montpellier, France; CREEC/CANECEV, MIVEGEC (CREES) Department, University of Montpellier, CNRS, IRD, Montpellier, France; Earth and Planets Laboratory, Carnegie Institution for Science, Washington, DC, USA; Institut de Cancérologie de l'Ouest-René Gauducheau, Centre de Recherche en Cancérologie, Saint Herblain, France; CREEC/CANECEV, MIVEGEC (CREES) Department, University of Montpellier, CNRS, IRD, Montpellier, France; Oncogenetic Department, University Hospital of Montpellier, Montpellier; European Liquid Biopsy Society (ELBS), Hamburg, Germany; CREEC/CANECEV, MIVEGEC (CREES) Department, University of Montpellier, CNRS, IRD, Montpellier, France; Laboratory of Rare Human Circulating Cells and Liquid Biopsy (LCCRH), University Medical Centre of Montpellier, France; Toulouse Biotechnology Institute University of Toulouse, INSA, CNRS, INRAE, Toulouse, France; Department of Biology, University of New Brunswick, Fredericton, NB, Canada

**Keywords:** cancer, evolution, selection for function, tumorigenesis

## Abstract

Tumorigenesis is widely studied within evolutionary frameworks, yet integrating genetic, non-genetic, spatial, and collective tumor-level dynamics into a unified description remains challenging. Here, we propose that tumorigenesis can be analyzed as a process in which tumors generate, explore, and filter phenotypic and organizational configurations over time. We use the concepts of phenotypic state-space exploration, selective funneling, and selection for function as an operational framework to describe how tumor systems diversify, become constrained, and stabilize or lose functional organization during cancer progression. In this view, tumors are not only populations of competing clones, but also dynamic systems whose persistence depends on plasticity, tissue constraints, spatial organization, and collective functional states. This perspective helps account for phenotypic plasticity, recurrent tumor architectures, and the emergence of collective properties such as group phenotypic composition. It also generates testable predictions, including convergence toward recurrent functional states, therapy-induced reorganization of accessible phenotypic space, and the disproportionate effect of disrupting key collective functions. By mapping these concepts onto tumor biology, we position cancer as a tractable system for studying constraint-driven tumor evolution and for informing therapeutic strategies that target tumor organization in addition to individual cancer cells.

## THE COMPLEXITY OF TUMOR EVOLUTION

Cancer is widely described as an evolutionary process [1–4], and this perspective has provided major insights into tumor initiation, progression, and therapeutic resistance. Over the past decades, evolutionary and ecological approaches have substantially expanded our understanding of cancer by incorporating clonal selection, intra-tumoral heterogeneity, and interactions with the microenvironment [5–9]. Importantly, contemporary evolutionary theory is sufficiently flexible to accommodate many of the complexities observed in tumors, including multi-level selection, phenotypic plasticity, and context-dependent fitness landscapes.

Nevertheless, the terminology and conceptual frameworks commonly used to describe cancer evolution do not always map straightforwardly onto a single, unified evolutionary description. For instance, the term “tumor evolution,” by analogy with expressions such as “human evolution,” could be interpreted as referring to evolutionary changes within tumor populations, with individual tumors acting as units of selection. In practice, however, it is most often used to describe the evolution of populations of tumor cells, in which individual cells constitute the primary units of selection. At the same time, the term is also widely used to encompass changes occurring at higher levels of organization, where tumors are viewed as societies of cells engaging in both competitive and cooperative interactions, giving rise to emergent properties at the level of cell groups or of the tumor as a whole. In this broader usage, tumor evolution refers to cumulative and constraint-biased changes in tumor organization over time, including changes in the spatial, ecological, and functional relationships among cell populations in response to microenvironmental selective pressures at the cell level. We use “constraint-biased” rather than “directional” to avoid implying an intrinsic tendency, goal, or progressive trajectory in tumor evolution towards a more “fit” tumor-level organizational state. The change observed in tumor progression is instead contingent and context-dependent: it results from the filtering of many generated variants and organizational states by tissue architecture, immune surveillance, vascular access, metabolic constraints, and therapeutic perturbations.

By referring to tumors as higher levels of organization, we do not mean that whole tumors generally behave as classical units of selection competing reproductively. Rather, a relevant higher level emerges when interactions among cancer cells, stromal cells, immune cells, vascular structures, extracellular matrix components, and soluble factors generate relatively stable collective configurations. These configurations can influence tumor persistence by maintaining spatial organization, metabolic complementarity, immune modulation, niche construction, or recovery after perturbation (see [10]). In this sense, the “higher level” is not itself a reproducing entity, but an emergent organizational state that can affect the persistence, recurrence, or progression of the entire tumor system.

These multiple usages of the term tumor evolution reflect both the complexity of tumor dynamics and the difficulty of integrating them within a single formal framework. Indeed, tumors are characterized by rapid genetic and phenotypic diversification as well as extensive plasticity at the cell level, but also by collective behaviors leading to emergent tumor-level properties that are not always easily reducible to clonal competition and selection based solely on heritable differences in reproductive cell fitness [11]. While selection undoubtedly operates at the level of cells and, in some contexts, cell clusters, whether tumors themselves can be considered units of selection with heritable variation in fitness remains debated [10, 12–14]. Moreover, although cell-level selection clearly occurs in many oncological contexts, particularly under therapeutic pressure, where resistant variants are favored [15–17], such selection typically operates within pre-structured phenotypic landscapes and does not, by itself, fully account for the emergence, stabilization, or recurrence of higher-level tumor organizations [10]. Examples of such higher-level organizations include spatially structured hypoxic or invasive niches, cooperative metabolic interactions between distinct cellular phenotypes, immune-modulatory tumor architectures, and therapy-induced resistant niches in which persistence depends on the organization of interacting cell populations rather than on the autonomous fitness of a single clone. We return to these examples below when discussing group phenotypic composition (GPC), selective funneling, metastasis, and therapeutic perturbation.

Recent conceptual advances have proposed complementary ways of describing evolutionary-like dynamics in systems where heritable variation in reproductive fitness among well-defined units is not always the most informative level of analysis. In particular, Wong *et al.* [18, 19] proposed a general framework that can be applied to both biotic and abiotic systems. In this perspective, differential reproductive success remains central to classical biological evolution, but additional forms of filtering may become relevant when the persistence of organizational states, rather than the reproductive success of a system, is under selection. Applied to tumor evolution, this framework highlights that collective tumor organizational states differ in their probability of persistence under local constraints. Tumor cell populations continuously generate alternative cellular and organizational states through mutation, plasticity, spatial rearrangement, and microenvironmental feedback. Most of these configurations are transient, unstable, or go extinct. Others persist because they maintain functions that support the tumor system, such as access to nutrients and oxygen, metabolic complementarity, immune modulation, stromal support, or recovery after therapeutic perturbation. This type of selection should therefore be understood as differential persistence of organizational states: configurations that provide and maintain such collective functions are more likely to remain detectable, re-emerge after perturbation, or contribute to progression, whereas configurations that fail to do so collapse or disappear.

Here, we aim to apply this evolutionary framework to cancer by focusing on situations in which cell lineage-based descriptions alone may be insufficient. Specifically, we propose that tumorigenesis can be understood as a process unfolding within an expanding phenotypic state space, structured by selective funneling and shaped by selection for system-level functions (discussed next). This perspective provides a unifying framework to account for genetic and non-genetic sources of variation, shifting levels of organization and selection, and the emergence of collective tumor properties. It also offers a basis for linking these concepts to observable features of tumor biology, as we outline below. Importantly, this framework does not simply re-describe existing evolutionary processes, but enables explicit integration of genetic, non-genetic, and collective dynamics within a single analytical perspective. In particular, it allows the identification of functional tumor states that are not reducible to clonal composition alone, and generates predictions about convergence, constraint, and reorganization at the tumor level that are difficult to formalize within cell lineage-centered approaches. As such, we believe that it provides an extended level of description that can be directly confronted with empirical data. More specifically, the added value relative to standard lineage-centered models is not that this framework invokes evolutionary mechanisms unknown to contemporary theory, but that it changes the object of analysis: alongside lineage frequencies and cell-level fitness, it tracks the accessibility, recurrence, and persistence of collective organizational states. This distinction is empirically meaningful only if such organizational states provide explanatory or predictive information beyond clonal genotype, clonal composition, or individual-cell phenotype. If they do not, the state-space/selective-funneling description would add no independent explanatory value.

## SELECTION FOR FUNCTION, STATE-SPACE EXPLORATION AND SELECTIVE FUNNELING: KEY CONCEPTS

Wong *et al.* [18] proposed that a broad class of systems can evolve if they exhibit three recurrent features: a set of interacting components capable of forming many possible configurations, processes that generate new configurations, and filtering or selection processes that preferentially retain configurations according to one or more functions related to static persistence, dynamic persistence, or novelty generation. We do not treat these features as necessary and sufficient conditions for evolution in the classical biological sense. Rather, we use them here as an operational lens for describing tumorigenesis (see next section) as a process in which cellular and microenvironmental components generate multiple phenotypic and organizational configurations, only a subset of which persist under tissue, immune, ecological, and therapeutic pressures.

More recently, Wong *et al.* [19] refined their general evolutionary framework on *selection for function* by introducing two interrelated mechanisms [19]: *state-space exploration* and *selective funneling*, whose combined effects result in state-space expansion. Together, these concepts describe how complex systems generate novelty through the exploration of possible configurations, and how only a restricted subset of these configurations persists under specific constraints. Importantly, in our use of this framework, these concepts do not replace classical biological evolution based on heritable variation in reproductive fitness. Instead, they provide an additional level of description for situations in which tumor behavior depends not only on clonal competition, but also on plasticity, tissue constraints, spatial organization, and collective tumor-level functions.

*State-space exploration* refers to the continual testing of the set of possible configurations a system can occupy (i.e. the *possibility spaces* that can contribute to the system’s function landscape). In biological systems, novel configurations arise through multiple mechanisms, including molecular innovation, phenotypic plasticity, stochastic fluctuations, developmental flexibility, and self-organizing interactions across levels of organization. However, an increasingly supported hypothesis is that non-hereditary cellular variability arises first (its own origins remaining an open question) and is subsequently stabilized by genetic and epigenetic changes, following a logic of “phenotypic first, genotypic second” change [20, 21]. Thus, state-space exploration is not restricted to gradual, mutation-driven change. It may involve abrupt transitions, combinatorial explosions of possibilities thanks to a continuum of phenotypes, or emergent properties that cannot be easily anticipated from prior states.

However, unrestricted exploration of a state space would overwhelmingly generate non-viable or transient configurations. This is where selective funneling plays a central role. Selective funneling describes how environmental constraints, boundary conditions, and internal system dynamics channel exploration toward a restricted subset of states that are compatible with persistence. These constraints can be physical, chemical, ecological, developmental, or interaction-based, and they operate regardless of whether classical reproduction-based selection is present. Selective funneling thus explains why, despite immense theoretical possibilities, biological systems repeatedly converge on a limited number of functional organizations. Importantly, funneling does not imply optimization toward a single peak; instead, it structures accessibility, stability, and recurrence within the explored state space.

Sometimes, a particular functional organization might open a new possibility space in a different dimension, whose functional landscape is characterized by different axes of relevance specific to the new behavior of the system. This shift is a new type of novelty, very different from intra-state-space exploration. Wong *et al.* [19] have referred to these events as state-space expansions and argued that every time a system transitions to a new higher level with new axes of relevance, a novel phenomenon emerges (see Wong *et al.* [19] for a more detailed discussion of the “emergence” concept). Notably, the “relevant axes of the new fitness landscape are descriptively and predictively accurate for any system that occupies that state space, regardless of how a system came to inhabit that space”; in other words, the systems exhibit multiple realizability. Furthermore, once entering the new possibility space, the system might be subjected to new driving forces for exploring combinatorial possibilities and new functional selective pressures, including shifts in units of selection. Such shifts can involve units above those associated with the Szathmáry and Smith’s major transitions in evolution [22] and extend to planetary-scale processes.

Wong *et al.* [19] intentionally developed this framework at a high level of abstraction, illustrating it with examples ranging from “evolution” in abiotic systems (e.g. isotopes in stars; minerals in planets) to the origin and evolution of life to cognition and social systems. This breadth is a major conceptual strength, as it suggests a unifying logic underlying diverse evolutionary phenomena. At the same time, such generality leaves open a critical question: how does this framework map onto concrete biological systems where mechanisms, constraints, and temporal dynamics are empirically observable? To date, applications of state-space expansion and selective funneling have remained largely conceptual, with few well-characterized systems explicitly analyzed through this lens.

## TUMORIGENESIS THROUGH THE LENS OF SELECTION FOR FUNCTION, STATE-SPACE EXPLORATION AND SELECTIVE FUNNELING

We have previously proposed that tumorigenesis can be understood within the framework of selection for function [10, 23]. Here, we argue that tumorigenesis can be explored through the expanded framework recently proposed by Wong *et al.* [19], across the continuum from early tumor development to rare but consequential outcomes such as metastasis. Tumors combine rapid and extensive phenotypic state-space exploration and expansion events with strong and recurrent selective funneling, leading to the stabilization, collapse, or reorganization of successive functional organizations within a single evolving system. Relative to many systems discussed in the context of state-space expansion and selective funneling, tumors offer distinct empirical advantages. Tumor evolution unfolds on observable timescales, occurs under well-characterized and often controllable perturbations such as therapy, and allows direct investigation of functional collapse, irreversibility, and reorganization. Moreover, emergent collective organization in tumors is increasingly, although still imperfectly, accessible through modern imaging, single-cell analyses, multi-region sampling, and spatial profiling approaches (e.g. [24–26]). In this sense, tumorigenesis should not be viewed as an exceptional or anomalous case, but rather as a tractable biological setting for analyzing how state-space exploration, selective funneling, and persistence-based filtering interact during tumor progression. We do not aim to provide an exhaustive analysis but instead explore tumor evolution through this framework, highlighting how successive functional organizations are stabilized or eliminated over time within a single tumor system. Previous contributions introduced selection for function as a general framework, applied persistence-based filtering to selected aspects of cancer evolution, or developed state-space expansion and selective funneling at a broad conceptual level. The specific contribution of the present manuscript is to bring these elements together in a single cancer-focused operational framework, map them onto measurable tumor properties—including GPCs, spatial architectures, interaction networks, and responses to perturbation—and derive empirical tests of whether this level of description adds predictive power beyond conventional clonal models.

In the tumor context, the relevant components capable of producing a variety of configurations include cancer cells, stromal cells, immune cells, vascular elements, extracellular matrix structures, and soluble signals. The generative processes include mutation, chromosomal instability, epigenetic change, transcriptional plasticity, stress-induced reprogramming, and microenvironmental feedback. The filtering processes include constraints imposed by tissue architecture, nutrient and oxygen availability, immune surveillance, therapy, and the requirement for collective persistence. Within this framework, functions are broadly understood as properties that contribute to system-level persistence, including static persistence, dynamic persistence, and the capacity to generate new configurations.

In classical evolutionary theory, selection acts through differential reproductive fitness among heritable variants, and this remains the central mechanism by which phenotypic change accumulates in populations of tumor cells. We do not argue that persistence or stability alone generates new phenotypes, nor that it replaces reproduction-based selection where this mechanism applies. Rather, in the framework used here, these alternative configurations arise through a range of genetic, epigenetic, phenotypic, spatial, and microenvironmental processes. Selection for function refers to the subsequent differential persistence, stabilization, or re-establishment of a subset of these configurations.

In this sense, selection for function should be understood as persistence-based filtering rather than as a substitute for Darwinian selection. It can bias the observed trajectory of tumor organization because configurations that maintain system-level functions, such as structural coherence, metabolic complementarity, immune modulation, vascular support, or recovery after perturbation, are more likely to persist, recur, or remain clinically detectable, whereas configurations that fail to maintain such functions disappear or collapse. The resulting change is therefore not directed in a teleological sense, nor does it require reproductive competition among whole tumors. It is a non-random filtering of generated variation that can operate alongside, and partly through, conventional clonal selection.

To avoid terminological ambiguity, we use the main concepts of this framework in an operational sense. By ``selection for function'', we mean the differential persistence, stabilization, or re-emergence of configurations that maintain functions contributing to tumor persistence. By “phenotypic state space,” we mean the set of cellular and organizational configurations that a tumor system can potentially occupy, including transcriptional, metabolic, spatial, ecological, and interaction-based states. By ``state-space exploration,'' we mean the generation or sampling of altemative configurations. By “state-space expansion,” we refer to situations in which new dimensions of variation become relevant to tumor behavior, e.g. when cellular heterogeneity gives rise to collective spatial organization, niche construction, invasion, or metastatic competence. Finally, by “selective funneling,” we mean the non-random restriction of this diversity by physiological, ecological, immune, spatial, or therapeutic constraints, such that only a subset of configurations persists. These terms are therefore used as analytical descriptors of tumor organization and persistence, not as replacements for classical evolutionary concepts such as heritable variation, reproductive fitness, or clonal selection.

We are aware that recent attempts to formulate very general principles of evolution and complexity have been criticized for overextending evolutionary theory (e.g. [27]), in particular when they imply a universal tendency toward increasing complexity. This is not the argument we make here. Our use of state-space exploration, selective funneling, and selection for function does not assume that evolution necessarily favors complexity, nor that tumor progression corresponds to a monotonic increase in complexity. Tumor evolution may involve diversification, simplification, collapse, loss of organization, or canalization toward a small number of recurrent states. Our claim is more limited: tumorigenesis provides a tractable biological system in which the generation of phenotypic and organizational variation, followed by strong filtering under physiological, ecological, and therapeutic constraints, can be empirically analyzed.

This framework also accommodates collapse, maladaptation, and evolutionary dead ends. Because state-space exploration generates many configurations, most of them will fail to satisfy the constraints required for persistence. Selective funneling therefore eliminates unstable or incompatible configurations as much as it stabilizes persistent ones. Moreover, persistence-based filtering is local and context-dependent rather than globally optimizing. A configuration that transiently supports growth, invasion, immune evasion, or therapy resistance may also create dependencies, deplete resources, increase genomic instability, reduce plasticity, or expose the tumor to later constraints. Such configurations may therefore become maladaptive when the microenvironment changes, when therapy is applied, or when internal organization can no longer be maintained. In this sense, collapse or evolutionary suicide can be understood as the failure of an explored configuration to maintain the collective functions required for continued tumor persistence.

## TUMORIGENESIS AS PHENOTYPIC STATE-SPACE EXPLORATION AND EXPANSION

In the context of the framework proposed by Wong *et al.* [19], we argue that tumorigenesis can be interpreted as an exceptionally rapid and extensive expansion of phenotypic state space (Fig. 1). Indeed, genetic and epigenetic instability, stochasticity in gene expression, metabolic rewiring, phenotypic plasticity, and continuous feedbacks with the microenvironment jointly generate an ever-growing repertoire of cellular states that collectively reflect in different tumor-level organizational states (e.g. [28–34]). Crucially, these new tumor-level states arise *de novo*, without direct descent from pre-existing tumors. As a result, tumor evolution (i.e. the change in its organizational landscape) cannot be adequately described as simply a change in the genetic make-up of the tumor cell population. Classical mutation-centered models, particularly those focusing on gradual, step-wise accumulation, may be insufficient on their own to fully capture macroevolutionary leaps, extensive phenotypic plasticity, and the profound influence of the microenvironment on cancer progression. Instead, the adaptive landscape in cancer is reshaped continuously by genetic and non-genetic mechanisms and complex interactions among clones and with a rapidly changing microenvironment, suggesting a dynamic, context-dependent “fitness seascape,” that is, a landscape that itself changes over time, rather than a static topography [6].

**Figure 1:**
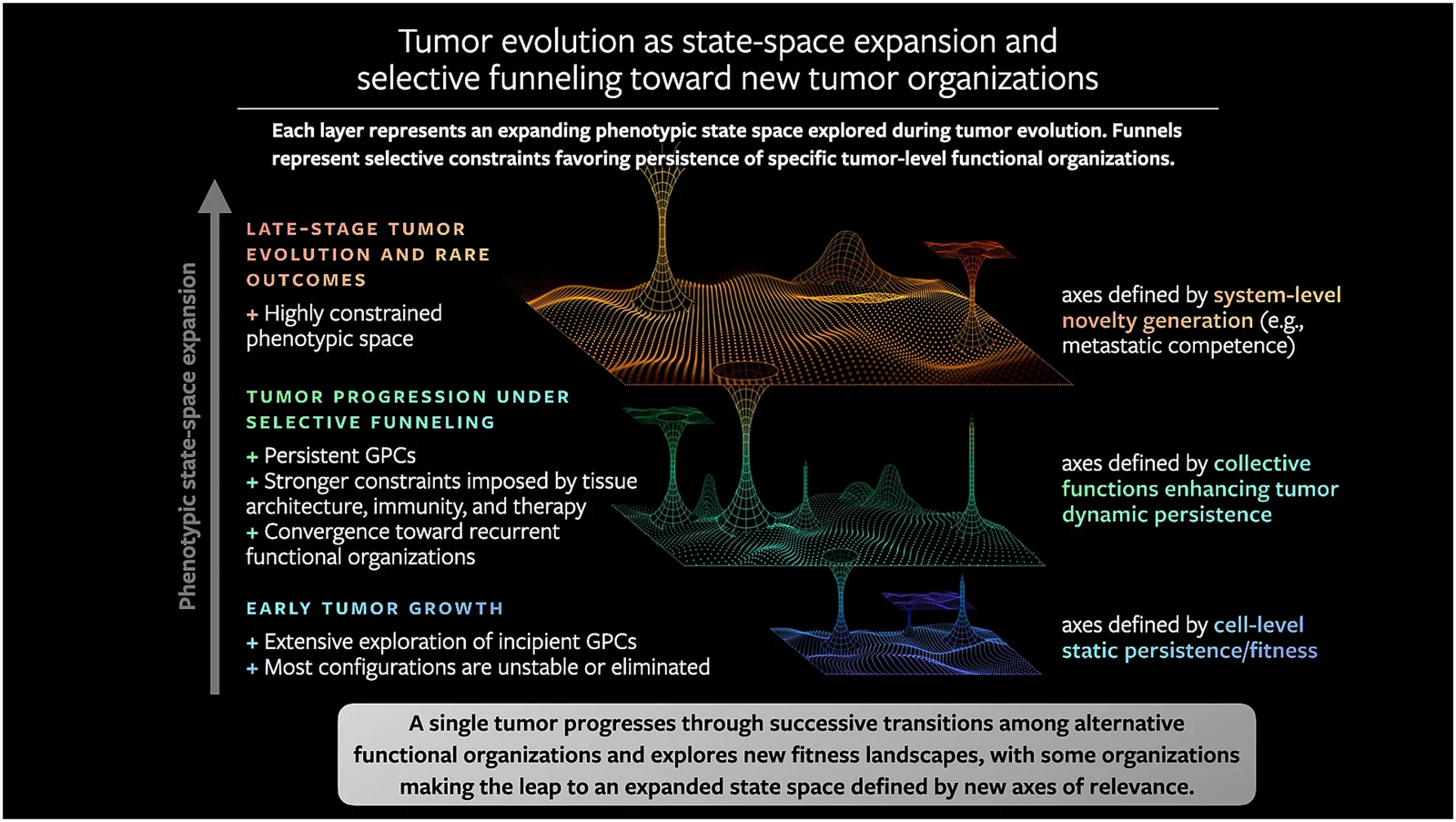
Tumorigenesis as an expanding phenotypic state space via selective funneling. Tumor evolution can be conceptualized as exploration of an existing possibility space followed by selective funneling toward a limited set of persistent tumor-level functional organizations, with occasional expansions into new higher-dimensional state spaces with different axes of relevance. Each horizontal layer represents a phenotypic state space accessible to the tumor at different stages of its evolution, shaped by genetic, epigenetic, ecological, and microenvironmental processes. The configuration of each landscape reflects the local stability of alternative configurations, while funnel-like structures represent selective constraints that restrict accessibility and favor persistence of specific organizational states. During early tumor evolution (bottom layer), extensive intra-state phenotypic exploration occurs under weak constraints, generating a wide diversity of transient cellular configurations. Most of these configurations are unstable or eliminated, but some achieve local functional stability, giving rise to incipient GPCs and pushing the tumor into a new dimensional state-space (central layer). As tumor progression unfolds (middle layer), selective funneling intensifies under constraints imposed by tissue architecture, immune interactions, and therapeutic perturbations. This process channels exploration toward recurrent and more stable GPCs characterized by collective functions that enhance tumor persistence, such as cooperative metabolism, spatial organization, and modulation of host interactions. Although local Darwinian selection may operate within these configurations, it remains embedded within broader system-level selection for function. At later stages, the accessible phenotypic space becomes highly constrained. Only rare configurations persist, corresponding to selective funneling towards a new state-space (top layer), with extreme outcomes such as metastatic competence and system-level robustness across heterogeneous environments. Metastasis is therefore depicted not as an inevitable endpoint of tumor evolution, but as a rare and highly filtered outcome of selective funneling. This abstract scenario depicts evolutionary changes that unfold within a single tumor through successive transitions among alternative functional organizations. This framework situates tumorigenesis as a tractable biological setting for analyzing state-space exploration, selective funneling, and selection for function. *This figure is an adaptation of the conceptual framework proposed by Wong et al.* [19] *adjusted here to the context of tumor evolution.*

This space exploration should not be interpreted as mere stochastic noise, but can instead be viewed as providing the raw material upon which selective funneling operates. Tumor cell populations continuously explore novel configurations of gene expression patterns, signaling pathways, metabolic strategies, motility programs, immune interactions, and collective organizational modes [10]. Importantly, this exploration occurs along two complementary dimensions. First, at any given time, tumors display substantial intra-tumoral heterogeneity, with multiple phenotypic (genetically or epigenetically) states coexisting within the same lesion. Second, exploration also unfolds sequentially over time within a single tumor, as the system transitions through successive organizational regimes. In this temporal sense, tumor evolution can be understood as a trajectory through an expanding state space, rather than as competition among static clones. At higher levels of organization, state-space expansion is not limited to individual cellular phenotypes but extends to group-level properties. Distinct spatial arrangements, cooperative interactions, and functional specializations among tumor cells give rise to alternative GPCs (see [11]). These GPCs represent emergent organizational states of the tumor (see Box 1), each characterized by specific collective functions, degrees of stability, and capacities for persistence. As the tumor evolves, it may repeatedly assemble, destabilize, and reassemble different GPCs, effectively sampling alternative regions of the expanded state space.

Box 1 GPC as a functional state of tumor organization.

GPC provides a concrete illustration of how the concepts of state-space exploration, selective funneling, and selection for function can be applied to tumor biology [11]. A GPC describes the relative abundance, spatial organization, and functional interactions among distinct cellular phenotypes within a tumor.

Within a state-space framework, each GPC corresponds to a position in a high-dimensional space defined by cellular states, interaction networks, and spatial configurations. Tumors continuously explore this space through genetic variation, phenotypic plasticity, and microenvironmental feedbacks, generating a wide diversity of possible organizational states.

Selective funneling is reflected in the recurrent observation that, despite extensive heterogeneity, tumors often converge toward a limited number of GPCs across patients and contexts [42–46]. This convergence suggests that only a restricted subset of configurations satisfies the constraints required for tumor persistence.

Selection for function is revealed by the repeated stabilization or re-emergence of specific GPCs following perturbations such as therapy. These configurations are not defined by the intrinsic fitness of individual clones, but by their contribution to system-level properties, including robustness, maintenance of heterogeneity, and adaptive reorganization.

This perspective generates testable predictions. For instance, tumors with distinct genetic trajectories may converge toward similar GPCs if subject to similar constraints. Conversely, therapeutic disruption of key functional interactions within a GPC should destabilize tumor organization, even if many cancer cells remain viable.

In this sense, GPCs provide a measurable proxy for functional states within the tumor state space, offering a tractable entry point for linking abstract evolutionary concepts to empirical observations and clinical outcomes.

At the cellular and genomic level, state-space exploration can be particularly abrupt. A striking illustration of non-gradual state-space exploration in cancer is chromothripsis, a phenomenon in which massive genomic rearrangements occur during a single catastrophic event [11, 35]. The triggers of chromothripsis remain incompletely understood. Available evidence indicates that chromothripsis may be initiated by intrinsic cellular mechanisms, including mitotic errors, telomere crisis, micronuclei formation, or replication stress, as well as by extrinsic DNA-damaging insults. In many cases, these catastrophic rearrangements appear to arise stochastically once cells are pushed beyond critical genomic stability thresholds. Thus, chromothripsis can generate, within a single catastrophic event, a large number of novel genetic configurations, dramatically enlarging the accessible phenotypic space in a manner that cannot be captured by incremental mutation-based models [36]. The vast majority of these configurations are non-viable or rapidly eliminated. This process partly overlaps with what would classically be described as purifying selection, insofar as many deleterious or non-viable variants are eliminated. However, selective funneling is used here in a broader and more organizational sense. Purifying selection typically refers to the removal of deleterious heritable variants from a population through differential reproductive fitness. By contrast, selective funneling refers to the non-random restriction of a much wider set of possible tumor configurations, including genetic, epigenetic, transcriptional, spatial, ecological, and collective organizational states. Some of these states may be transient, plastic, or interaction-dependent rather than strictly heritable lineages. Thus, purifying selection can be viewed as one local mechanism contributing to selective funneling, but selective funneling also includes constraints that act on accessibility, stability, recurrence, and persistence of tumor-level organizations. However, a small subset may fortuitously fall within regions of the expanded state space that support emergent properties at the tumor level. In this case, innovation precedes selection: novelty is generated first, and selective funneling subsequently constrains which configurations persist. While chromothripsis operates at the level of individual genomes, its evolutionary significance lies in how a small subset of the resulting cellular states may later participate in higher-level organizations of the tumor. This is a clear example of state-space expansion whose functional landscape is defined by new axes of relevance that better describe the system’s behavior.

More generally, evidence suggests that tumorigenesis often involves episodes in which innovation is decoupled from immediate selective optimization (e.g. [8]). Plasticity-driven state switching, stress-induced reprogramming, and microenvironmentally induced phenotypes can all generate functional states that are not strictly heritable in a lineage-based sense [37, 38]. Yet these states may recur, stabilize, and contribute to tumor persistence if they align with funneling constraints. This decoupling highlights a key departure from classical individual-level fitness expectations and underscores why tumor evolution is particularly well suited to a state-space expansion perspective.

This argument is closely related to niche construction theory, in which organisms modify their own selective environments and thereby alter subsequent evolutionary dynamics [39, 40]. In tumors, cancer cells and associated stromal, immune, and vascular components can similarly modify their local microenvironment through extracellular matrix remodeling, angiogenesis, metabolic waste production, immune modulation, and pre-metastatic niche formation. These processes can change the constraints under which later tumor configurations are generated and filtered. We therefore view niche construction as one important mechanism contributing to selective funneling in cancer. However, the state-space framework is broader in scope: it also includes configurations generated by intrinsic instability, plasticity, spatial rearrangement, therapy-induced perturbation, and collective organization, even when these do not involve active modification of the environment by tumor cells. Thus, niche construction helps explain how tumors reshape parts of their selective context, whereas selective funneling describes the broader restriction of possible cellular and organizational states under both internally generated and externally imposed constraints.

In summary, tumorigenesis, in our view, exemplifies an evolutionary process in which continuous and sometimes abrupt exploration of phenotypic state space precedes and enables selective structuring. Rather than evolving solely through differential replication of pre-defined variants, tumors generate and explore novel organizational possibilities, setting the stage for selective funneling toward a limited set of persistent functional configurations. Importantly, empirical observations in oncology are inherently biased toward tumors that succeed in achieving such persistence. Clinically detectable cancers therefore represent the outcome of strong filtering processes, in which only a small subset of possible tumor trajectories, those that reach functionally stable organizations, remain observable. This observational bias is an important limitation, but it does not make the framework unfalsifiable. It means that the full distribution of generated configurations cannot usually be reconstructed from clinically detectable tumors alone. Consequently, we do not claim that the source of variation can always be assigned unambiguously to a single process, whether Darwinian, plastic, environmentally induced, or saltatory. Rather, the framework generates testable expectations about the relationship between variation generation, filtering, and persistence. For example, it predicts that prospective longitudinal sampling, experimental tumor models, lineage tracing, organoids, and perturbation experiments should reveal a broader range of transient phenotypic and organizational states than those observed among persistent tumors; that this diversity should become non-randomly restricted under recurrent constraints; and that the configurations that persist or re-emerge after perturbation should be enriched for collective functions supporting tumor persistence. Conversely, the framework would be weakened if transient variation were not broader than persistent variation, if filtering under similar constraints did not produce recurrent organizational states, or if such states failed to predict persistence, relapse, or metastatic competence beyond clonal genotype alone.

## SELECTIVE FUNNELING IN TUMOR EVOLUTION

*Despite vast phenotypic and genetic diversity, tumors often appear to converge on a limited set of organizations* [41]; that is, they exhibit *multiple realizability.* An important alternative, and in many cases complementary, explanation is that repeatability in tumor evolution may arise from a many-to-one genotype–phenotype map coupled with classical Darwinian selection. Under this view, genetically heterogeneous clones may converge toward similar cellular phenotypes because different genetic trajectories produce equivalent functional outputs, which are then favored by differential reproductive fitness. We fully acknowledge that this mechanism can account for many cases of phenotypic convergence in cancer. Our argument is not that selective funneling replaces this explanation, but that it extends the level at which repeatability can be analyzed. The relevant question is whether convergence is observed only at the level of individual cellular phenotypes, or also at the level of multicellular organization, including GPCs, spatial architectures, interaction networks, and therapy-induced resistant niches. Evidence that such organizational states predict persistence, relapse, or metastatic competence beyond clonal genotype or individual-cell phenotype alone would support the added value of the state-space and selective-funneling perspective. *From a selective funneling perspective, this convergence can be interpreted as reflecting constraints imposed by tissue architecture, immune surveillance, vascularization, and therapeutic interventions,* which act as powerful filters channeling state-space exploration *toward a restricted subset of organizational states compatible with persistence* (e.g. [42, 43]) (Fig. 1). *Such constraints likely contribute to the recurrence of similar tumor organizations across organs, individuals, and even species, despite extensive genetic and contextual heterogeneity* [44–46].

In addition to acting through individual clones, selective funneling can also be reflected in the stabilization of collective functional architectures, including cooperative metabolic networks, spatial patterning of phenotypes, and coordinated invasive behaviors. These architectures correspond to distinct GPCs, which may emerge, destabilize, and re-emerge sequentially during the lifetime of a single tumor [10]. This convergence through self-organization accounts for why genetically distinct tumors can nonetheless display remarkably similar functional organizations and shows that selective funneling can operate entirely within a single tumor, without requiring tumor “reproduction” and selection among tumor entities.

Therapeutic interventions represent particularly strong funneling forces. While therapies often induce selection at the cellular level by favoring resistant clones with heritable survival advantages, this clonal selection does not operate in isolation. Instead, we argue that it is embedded within higher-level funneling processes that stabilize specific functional configurations of the tumor system, such as resistant niches, cooperative metabolic states, or phenotypically heterogeneous assemblies. In this sense, clonal selection acts as a local mechanism within a globally constrained and functionally structured evolutionary process (see also [10]).

We also propose here that metastasis provides an extreme illustration of selective funneling in tumor evolution and a direct manifestation of state-space expansion and selection for function. Although tumor cells may disseminate in large numbers, only an extremely small subset succeed in establishing secondary lesions, indicating that dissemination alone is insufficient for persistence [47]. From a state-space expansion perspective, metastatic progression corresponds to the exploration of a new exceptionally constrained region of phenotypic space, where only rare configurations are viable. Crucially, metastatic success need not be explained exclusively either by the intrinsic fitness of individual disseminated cells or by higher-level functional organization. Individual disseminated cells may possess traits that enhance survival, motility, plasticity, immune evasion, or colonization capacity. However, these traits often become effective only within broader organizational and ecological contexts, including niche preconditioning, self-seeding, collective interactions, stromal support, immune modulation, and the capacity to exploit or modify distant microenvironments. In this sense, functional organization and individual-cell fitness are complementary rather than mutually exclusive explanations of metastatic success [23]. Metastasis should therefore be understood not as an inevitable endpoint of tumorigenesis, but as a rare yet highly structured functional outcome emerging when selective funneling aligns cellular plasticity, environmental constraints, and system-level organizations that confer robustness to the tumor system across contexts [37].

## SELECTION WITHOUT REPRODUCTION: PERSISTENCE AS THE TARGET OF SELECTION

In tumors, “fitness” in the classical Darwinian sense is often difficult to define at the level of the tumor as a whole. Cell division rates, survival probabilities, and reproductive success vary across space, time, and microenvironmental context. What remains consistent, however, is tumor persistence, that is, the ability of the tumor as an integrated complex system to maintain itself over time. Importantly, persistence-based filtering should not be understood as persistence alone. As in classical selection, enrichment among observed survivors results from both the persistence of some configurations and the elimination, collapse, or disappearance of others. In this respect, the concept of selection for tumor persistence is consistent with Doolittle’s concept of “natural selection for survival alone,” based on the fact that “differential survival of non-competing and non-reproducing individuals will also result in increasing frequencies of survival-promoting ‘adaptations’ among survivors” [48]. This concept has also been used to reconcile Gaia with evolutionary theory by arguing that although there is only one biosphere, and reproductive competition among biospheres cannot take place, survival-promoting configurations can nevertheless become enriched among what persists [48].

This logic partly resembles purifying selection, because unstable, deleterious, or non-persistent configurations are removed from the observable set. However, in the context considered here, what is filtered is not only heritable cellular variants through differential reproductive fitness, but also transient, plastic, spatial, and collective tumor configurations through their differential capacity to persist, re-emerge, or collapse under local constraints. Accordingly, what is under filtering is not only the stable phenotypes of individual cells, but also the phenotypic composition and functional organization of the tumor as a whole.

Functions subject to selection for tumor persistence include the maintenance of cooperative heterogeneity, regulation of interactions with the microenvironment, evasion or manipulation of host responses, and the capacity to reorganize following perturbations [11]. These functions typically emerge at the tumor level and need not be heritable in a classical lineage-based sense. Their repeated stabilization and re-emergence are consistent with persistence-based filtering of tumor-level functional organization, operating alongside individual cell-level fitness differences. At the level of individual cell lineages, persistence-based and classical fitness-based descriptions may often make overlapping predictions because lineage persistence is achieved largely through differential replication. Their distinction becomes informative when persistence depends on collective, spatial, or dynamical properties that are not reducible to the reproductive success of any single lineage. Tumors as wholes, however, generally do not exhibit heritable variation in fitness (see [10] for discussion), and their persistence frequently depends on such higher-level properties; in these cases, classical cell-level fitness alone may be an incomplete descriptor of what is being filtered.

### From conceptual framework to measurable parameters

A key challenge for any general evolutionary framework is its ability to be translated into measurable parameters and testable predictions. While the concepts of state-space exploration, selective funneling, and selection for function are inherently abstract, they can be operationalized using existing data types and analytical approaches in cancer biology. The goal of the present study is to establish a conceptual and operational foundation that enables future empirical validation of this framework. By explicitly linking theoretical constructs to measurable parameters and existing data types, we aim to facilitate empirical and modeling studies designed to evaluate the relevance and predictive power of this approach in concrete oncological systems.

**(i) State-space exploration and expansion** can be approximated by quantifying the diversity, accessibility, and temporal dynamics of phenotypic configurations within a tumor, ideally using longitudinal or multi-region data to capture trajectories through phenotypic space. Metrics such as phenotypic entropy, dispersion, or manifold volume can serve as proxies for the extent of explored state space. Longitudinal or multi-region datasets further allow reconstruction of trajectories through this space, providing insight into whether tumors expand into novel regions or remain confined to pre-existing configurations.

**(ii) Selective funneling** can be inferred from patterns of convergence, constraint, and recurrence within this phenotypic space. For instance, despite high initial heterogeneity, tumors often converge toward a limited number of transcriptional or functional states. This convergence can be quantified using clustering stability, attractor analysis, or reductions in phenotypic variance over time or across patients. Spatial transcriptomics and imaging approaches additionally allow identification of recurrent spatial organizations, providing a direct measure of constraints acting on tumor architecture. In this context, funneling is expected to manifest as a non-random restriction of accessible states, even in the presence of ongoing variability.

**(iii) Selection for function** can be approached by identifying phenotypic configurations that contribute to tumor persistence rather than short-term proliferative success alone. In practice, this may involve linking specific GPCs (see Box 1), spatial arrangements, or interaction networks to clinically relevant outcomes such as stability under therapy, relapse dynamics, or metastatic success. Network-based analyses, perturbation experiments, and longitudinal monitoring can help identify which configurations are repeatedly stabilized or re-established following perturbations, thus revealing the functional targets of selection.

The connection between these concepts and empirical predictions follows from the sequential logic of the framework. If tumorigenesis involves broad phenotypic state-space exploration, then tumors should initially generate a larger diversity of cellular and organizational states than those ultimately observed among persistent tumors. If selective funneling operates, then this diversity should become non-randomly restricted by recurrent constraints such as tissue architecture, immune pressure, vascular access, metabolic limitation, and therapy. If selection for function contributes to tumor persistence, then the configurations that are repeatedly stabilized should be those that support collective properties such as robustness, spatial organization, metabolic cooperation, immune modulation, or recovery after perturbation. These links generate several testable predictions.

First, while convergence itself is a well-established expectation of evolutionary theory, our framework shifts the predicted level of convergence from individual cellular traits or genotypes to recurrent functional and organizational states of the tumor system. Tumors exposed to similar constraints should therefore converge not only toward similar cellular phenotypes, but also toward similar GPCs, spatial architectures, interaction networks, or transcriptional attractor states, even when their underlying genetic trajectories differ. This prediction follows from selective funneling: if constraints restrict the accessible phenotypic space, different genotypes may nevertheless be channeled toward similar GPCs, spatial architectures, or transcriptional attractor states. This can be tested by comparing multi-region or single-cell datasets across tumors with distinct mutational histories but similar tissues, microenvironments, or treatment pressures.

Second, therapeutic interventions should reshape not only clonal composition but also the accessible phenotypic and organizational space of the tumor. Mechanistically, therapy can remove some cellular states, create vacant ecological niches, alter stromal and immune interactions, modify oxygen and nutrient gradients, induce stress responses and plasticity programs, and change the selective value of pre-existing phenotypes. Therapy therefore acts not only as a selective pressure on resistant clones, but also as a reconfiguration of the constraints that determine which phenotypic and organizational states remain accessible. Under similar therapeutic constraints, this may lead to recurrent resistant niches, phenotypically heterogeneous assemblies, or alternative GPCs. This can be tested by longitudinally comparing pre-treatment, residual, and relapse samples using single-cell, spatial, and functional profiling.

Third, perturbations that disrupt key collective functions should have disproportionate effects on tumor persistence relative to their effects on individual cancer cell viability. This prediction follows from selection for function: if tumor persistence depends on system-level organization, then disrupting the interactions that maintain a GPC, such as metabolic cooperation, immune suppression, vascular support, or spatial compartmentalization, should destabilize the tumor even when many individual cells remain viable. This can be tested experimentally by targeting interaction networks or microenvironmental dependencies and measuring organizational collapse, relapse probability, or loss of metastatic competence.

These predictions also specify conditions under which the framework would be weakened or rendered unnecessary. It would add little beyond standard lineage-centered models if transient organizational diversity were not broader than persistent diversity; if comparable constraints did not reproducibly narrow accessible state space or yield recurrent organizational states; if GPCs, spatial architectures, or interaction networks failed to predict persistence, relapse, or metastatic competence beyond genotype and individual-cell phenotype; or if disrupting collective interactions had no effect on tumor persistence beyond effects attributable to individual-cell viability. Under such outcomes, conventional clonal evolutionary models would provide the more parsimonious description.

An important limitation is that these approaches currently provide imperfect proxies rather than complete reconstructions of tumor organizational states. This limitation is partly technological: spatial resolution, sampling depth, temporal coverage, tissue preservation, and the integration of molecular, spatial, and functional information remain constrained. It is also partly conceptual, because identifying a GPC or a functional organizational state requires more than detecting cell types or transcriptional clusters; it requires inferring interactions, spatial dependencies, and contributions to persistence. For this reason, the framework should not be interpreted as assuming that such states can already be fully observed in routine datasets. Rather, existing technologies allow partial and testable approximations that can be strengthened by combining longitudinal sampling, perturbation experiments, spatial profiling, single-cell analyses, imaging, and functional validation.

### Therapeutic implications

Viewing tumorigenesis through the lens of selective funneling and state-space expansion fundamentally reframes therapeutic objectives. Rather than aiming to eradicate all malignant cells, effective interventions may instead target the structural and functional constraints that stabilize persistent tumor organizations. In this perspective, therapy seeks to disrupt pathways to novelty generation, destabilize collective tumor functions, and drive the system beyond critical tipping points where functional reorganization becomes impossible (see [49] for a recent review).

Importantly, this framework suggests that many of the most successful current therapies already operate in this manner, often without being explicitly designed to do so [49]. Indeed, a growing body of evidence indicates that durable tumor control frequently arises not from maximal cytotoxicity, but from the destabilization of the tumor’s GPC, that is, the collective organization, spatial structure, and cooperative networks that sustain malignant persistence. By “functional organization,” we do not refer to a separate entity superimposed on the tumor, but to a relational and dynamic state of the tumor system. Such an organization emerges when particular cellular phenotypes, spatial arrangements, signaling interactions, stromal dependencies, immune-modulatory effects, and resource flows become coupled in ways that support persistence. It is maintained over time through feedbacks among tumor cells and their microenvironment, including reciprocal signaling, niche modification, metabolic complementarity, vascular support, immune modulation, and the repeated re-establishment of spatially structured interactions after perturbation. Thus, a functional organization is not defined by the mere presence of specific cell types, but by the persistence of interactions among them that sustain tumor-level properties such as robustness, recovery, invasion, or resistance. When therapeutic perturbations push this organization beyond a functional threshold, the tumor collective can lose coherence and collapse, even if a substantial fraction of tumor cells remains viable.

Such strategies naturally align with adaptive and system-level therapeutic approaches, which focus on controlling, reshaping, or destabilizing tumor organization rather than maximizing short-term cell killing (see [49]). By targeting the functional architecture of tumors instead of individual cells, this perspective unifies diverse therapeutic paradigms, including immune-mediated strategies, adaptive dosing, differentiation therapy, and microenvironmental normalization, under a common principle: durable control emerges from the breakdown of collective functionality rather than from the elimination of every malignant cell.

## CONCLUSIONS

Tumorigenesis provides a concrete biological setting in which concepts such as phenotypic state-space exploration, selective funneling, and selection for function can be operationalized and tested. Rather than treating these concepts as a new universal law of evolution, we use them as a complementary analytical framework for describing how tumor systems generate variation, undergo constraint-driven filtering, and stabilize or lose functional organization over time. By explicitly mapping cancer evolution onto the concepts of state-space exploration/expansion and selective funneling, we suggest that tumors provide a useful empirical setting for analyzing how cell-level selection, phenotypic plasticity, spatial organization, and persistence-based filtering interact during cancer progression. Crucially, in tumors, evolutionary change can affect both cancer cell populations (clonal evolution; change in genetic make-up) as well as the tumor itself as a self-organizing complex system whose organizational state can change through temporal transitions among alternative functional organizations. Notably, empirical observations in oncology are necessarily biased toward tumors that succeed in persisting and progressing. Within a selective funneling framework, clinically detectable cancers can be understood as the outcome of strong filtering processes, whereby only a small subset of possible tumor trajectories, those achieving functionally stable organizations, remain observable.

This perspective provides a complementary framework to clarify what is selected, at which level, and under which constraints. More broadly, it positions tumors as valuable empirical systems for refining evolutionary descriptions of cancer in cases where lineage-centered explanations alone do not capture the full dynamics of tumor organization. Rather than opposing Darwinian and non-Darwinian views of cancer evolution, our approach places them within a common descriptive framework, in which clonal selection remains a central process while state-space exploration and constraint-driven filtering capture organizational dynamics that may not be reducible to lineage change alone.

## References

[1] Cairns J . Mutation selection and the natural history of cancer. *Nature* 1975;255:197–200. 10.1038/255197a0 1143315

[2] Nowell PC . The clonal evolution of tumor cell populations. *Science* 1976;194:23–8. 10.1126/science.959840 959840

[3] Merlo LMF, Pepper JW, Reid BJ et al. Cancer as an evolutionary and ecological process. *Nat Rev Cancer* 2006;6:924–35. 10.1038/nrc2013 17109012

[4] Greaves M, Maley CC. Clonal evolution in cancer. *Nature* 2012;481:306–13. 10.1038/nature10762 PMC3367003 22258609

[5] Laplane L, Maley CC. The evolutionary theory of cancer: challenges and potential solutions. *Nat Rev Cancer* 2024;24:718–33. 10.1038/s41568-024-00734-2 PMC11627115 39256635

[6] Vendramin R, Litchfield K, Swanton C. Cancer evolution: Darwin and beyond. *EMBO J* 2021;40:e108389. 10.15252/embj.2021108389 PMC8441388 34459009

[7] Wang HY, Chen Y, Tong D et al. Is the evolution in tumors Darwinian or non-Darwinian? *Natl Sci Rev* 2018;5:15–7. 10.1093/nsr/nwx076

[8] Ling S, Hu Z, Yang Z et al. Extremely high genetic diversity in a single tumor points to prevalence of non-Darwinian cell evolution. *Proc Natl Acad Sci USA* 2015;112:E6496–505. 10.1073/pnas.1519556112 PMC4664355 26561581

[9] Huang S, Soto AM, Sonnenschein C. The end of the genetic paradigm of cancer. *PLoS Biol* 2025;23:e3003052. 10.1371/journal.pbio.3003052 PMC12136056 40100793

[10] Thomas F, DeGregori J, Marusyk A et al. A new perspective on tumor progression: evolution via selection for function. *Evol Med Public Health* 2024;12:172–7. 10.1093/emph/eoae021 PMC11448472 39364294

[11] Capp J-P, DeGregori J, Nedelcu AM et al. Group phenotypic composition in cancer. *eLife* 2021;10:e63518. 10.7554/eLife.63518 PMC8009660 33784238

[12] Greaves M . Cancer stem cells as 'units of selection'. *Evol Appl* 2013;6:102–8. 10.1111/eva.12017 PMC3567475 23396760

[13] Okasha S . Cancer and the levels of selection. *Br J Philos Sci* 2024;75:537–60. 10.1086/716178

[14] Laplane L, Lamoureux A, Richker HI et al. Applying multilevel selection to understand cancer evolution and progression. *PLoS Biol* 2025;23:e3003290. 10.1371/journal.pbio.3003290 PMC12303392 40680084

[15] Gerlinger M, Swanton C. How Darwinian models inform therapeutic failure initiated by clonal heterogeneity in cancer medicine. *Br J Cancer* 2010;103:1139–43. 10.1038/sj.bjc.6605912 PMC2967073 20877357

[16] Álvarez-Arenas A, Podolski-Renic A, Belmonte-Beitia J et al. Interplay of Darwinian selection, Lamarckian induction and microvesicle transfer on drug resistance in cancer. *Sci Rep* 2019;9:9332. 10.1038/s41598-019-45863-z PMC6597577 31249353

[17] Belkhir S, Thomas F, Roche B. Darwinian approaches for cancer treatment: benefits of mathematical modeling. *Cancers (Basel)* 2021;13:4448. 10.3390/cancers13174448 PMC8431137 34503256

[18] Wong ML, Cleland CE, Arend D Jr et al. On the roles of function and selection in evolving systems. *Proc Natl Acad Sci USA* 2023; 120:e2310223120. 10.1073/pnas.2310223120 PMC10614609 37844243

[19] Wong ML, Demarest H, Bartlett S et al. Selective funnelling and state-space expansion: a conceptual framework for novelty generation and origin events. *Interface Focus* 2025;15:20250009. 10.1098/rsfs.2025.0009

[20] Capp J-P . Tissue disruption increases stochastic gene expression thus producing tumors: Cancer initiation without driver mutation. *Int J Cancer* 2017;140:2408–13. 10.1002/ijc.30596 28052327

[21] Frank SA, Yanai I. The origin of novel traits in cancer. *Trends Cancer* 2024;10:880–92. 10.1016/j.trecan.2024.07.005 39112299

[22] Szathmáry E, Smith JM. The major evolutionary transitions. *Nature* 1995;374:227–32. 10.1038/374227a0 7885442

[23] Thomas F, Dujon AM. Beyond multilevel selection in cancer: rethinking metastasis through selection for function. *BioEssays* 2026;48:e70094. 10.1002/bies.70094 PMC12706130 41340363

[24] Papalexi E, Satija R. Single-cell RNA sequencing to explore immune cell heterogeneity. *Nat Rev Immunol* 2018;18:35–45. 10.1038/nri.2017.76 28787399

[25] Marusyk A, Almendro V, Polyak K. Intra-tumour heterogeneity: a looking glass for cancer? *Nat Rev Cancer* 2012;12:323–34. 10.1038/nrc3261 22513401

[26] Larsson L, Frisén J, Lundeberg J. Spatially resolved transcriptomics adds a new dimension to genomics. *Nat Methods* 2021;18:15–8. 10.1038/s41592-020-01038-7 33408402

[27] Lynch M . Complexity myths and the misappropriation of evolutionary theory. *Proc Natl Acad Sci USA* 2025;122:e2425772122. 10.1073/pnas.2425772122 PMC12167962 40408411

[28] Guo S, Zhu X, Huang Z et al. Genomic instability drives tumorigenesis and metastasis and its implications for cancer therapy. *Biomed Pharmacother* 2023;157:114036. 10.1016/j.biopha.2022.114036 36436493

[29] Bhat GR, Sethi I, Sadida HQ et al. Cancer cell plasticity: from cellular, molecular, and genetic mechanisms to tumor heterogeneity and drug resistance. *Cancer Metastasis Rev* 2024;43:197–228. 10.1007/s10555-024-10172-z PMC11016008 38329598

[30] Ng CX, Lee SY, Yap XY et al. Epigenetic reprogramming as the nexus of cancer stemness and therapy resistance: implications for biomarker discovery. *Discov Oncol* 2025;16:2220. 10.1007/s12672-025-04085-8 PMC12738525 41258574

[31] Xu X, Peng Q, Jiang X et al. Metabolic reprogramming and epigenetic modifications in cancer: from the impacts and mechanisms to the treatment potential. *Exp Mol Med* 2023;55:1357–70. 10.1038/s12276-023-01020-1 PMC10394076 37394582

[32] Laisné M, Lupien M, Vallot C. Epigenomic heterogeneity as a source of tumour evolution. *Nat Rev Cancer* 2025;25:7–26. 10.1038/s41568-024-00757-9 39414948

[33] Hinohara K, Polyak K. Intratumoral heterogeneity: more than just mutations. *Trends Cell Biol* 2019;29:569–79. 10.1016/j.tcb.2019.03.003 PMC6579620 30987806

[34] Lawson DA, Kessenbrock K, Davis RT et al. Tumour heterogeneity and metastasis at single-cell resolution. *Nat Cell Biol* 2018;20:1349–60. 10.1038/s41556-018-0236-7 PMC6477686 30482943

[35] Cortés-Ciriano I, Lee JJK, Xi R et al. Comprehensive analysis of chromothripsis in 2,658 human cancers using whole-genome sequencing. *Nat Genet* 2020;52:331–41. 10.1038/s41588-019-0576-7 PMC7058534 32025003

[36] Krupina K, Goginashvili A, Cleveland DW. Scrambling the genome in cancer: causes and consequences of complex chromosome rearrangements. *Nat Rev Genet* 2024;25:196–210. 10.1038/s41576-023-00663-0 PMC10922386 37938738

[37] Zaarour RF, Ribeiro M, Azzarone B et al. Tumor microenvironment-induced tumor cell plasticity: relationship with hypoxic stress and impact on tumor resistance. *Front Oncol* 2023;13:1222575. 10.3389/fonc.2023.1222575 PMC10598765 37886168

[38] Gantner BN, Palma FR, Pandkar MR et al. Metabolism and epigenetics: drivers of tumor cell plasticity and treatment outcomes. *Trends Cancer* 2024;10:992–1008. 10.1016/j.trecan.2024.08.005 39277448

[39] Odling-Smee J, Erwin DH, Palkovacs EP et al. Niche construction theory: a practical guide for ecologists. *Q Rev Biol* 2013;88:4–28. 10.1086/669266 23653966

[40] Laland K, Matthews B, Feldman MW. An introduction to niche construction theory. *Evol Ecol* 2016;30:191–202. 10.1007/s10682-016-9821-z PMC4922671 27429507

[41] Hanahan D, Weinberg RA. Hallmarks of cancer: the next generation. *Cell* 2011;144:646–74. 10.1016/j.cell.2011.02.013 21376230

[42] Giraudeau M, Sepp T, Ujvari B et al. Differences in mutational processes and intra-tumour heterogeneity between organs: the local selective filter hypothesis. *Evol Med Public Health* 2019;2019:139–46. 10.1093/emph/eoz017 PMC6735757 31528343

[43] Pienta KJ, Hammarlund EU, Axelrod R et al. Convergent evolution, evolving evolvability, and the origins of lethal cancer. *Mol Cancer Res* 2020;18:801–10. 10.1158/1541-7786.MCR-19-1158 PMC7272288 32234827

[44] Chen H, He X. The convergent cancer evolution toward a single cellular destination. *Mol Biol Evol* 2016;33:4–12. 10.1093/molbev/msv212 26464125

[45] Somarelli JA, Gardner H, Cannataro VL et al. Molecular biology and evolution of cancer: from discovery to action. *Mol Biol Evol* 2020;37:320–6. 10.1093/molbev/msz242 PMC6993850 31642480

[46] Boddy AM, Harrison TM, Abegglen LM. Comparative oncology: new insights into an ancient disease. *iScience* 2020;23:101373. 10.1016/j.isci.2020.101373 PMC7394918 32738614

[47] Tissot T, Massol F, Ujvari B et al. Metastasis and the evolution of dispersal. *Proc Biol Sci* 2019;286:20192186. 10.1098/rspb.2019.2186 PMC6939260 31771479

[48] Doolittle WF . Darwinizing Gaia: Natural Selection and Multispecies Community Evolution. Cambridge, MA: MIT Press, 2024.

[49] Thomas F, Capp J-P, Dujon AM et al. Leveraging selection for function in tumor evolution: system-level cancer therapies. *Evol Med Public Health* 2025;13:248–68. 10.1093/emph/eoaf022 PMC12448390 40979445

